# Special delivery: distributing iron in the cytosol of mammalian cells

**DOI:** 10.3389/fphar.2014.00173

**Published:** 2014-07-22

**Authors:** Caroline C. Philpott, Moon-Suhn Ryu

**Affiliations:** Genetics and Metabolism Section, Liver Diseases Branch, National Institute of Diabetes and Digestive and Kidney Diseases, National Institutes of HealthBethesda, MD, USA

**Keywords:** non-heme iron, diiron, glutathione, metallochaperone, iron chaperone, glutaredoxin, labile iron pool

## Abstract

Eukaryotic cells contain hundreds of proteins that require iron cofactors for activity. These iron enzymes are located in essentially every subcellular compartment; thus, iron cofactors must travel to every compartment in the cell. Iron cofactors exist in three basic forms: Heme, iron–sulfur clusters, and simple iron ions (also called non-heme iron). Iron ions taken up by the cell initially enter a kinetically labile, exchangeable pool that is referred to as the labile iron pool. The majority of the iron in this pool is delivered to mitochondria, where it is incorporated into heme and iron–sulfur clusters, as well as non-heme iron enzymes. These cofactors must then be distributed to nascent proteins in the mitochondria, cytosol, and membrane-bound organelles. Emerging evidence suggests that specific systems exist for the distribution of iron cofactors within the cell. These systems include membrane transporters, protein chaperones, specialized carriers, and small molecules. This review focuses on the distribution of iron ions in the cytosol and will highlight differences between the iron distribution systems of simple eukaryotes and mammalian cells.

One of the most important concepts to emerge from the field of eukaryotic cell biology is that the contents of the cell are highly organized and the movement of organelles and macromolecules within the cell does not occur through simple diffusion. Movement is highly regulated through packaging and assembly of complexes and through directed transport via components of the cytoskeleton. Cells express hundreds, perhaps thousands of proteins that require bound metal cofactors for activity and stability (Waldron et al., 2009). Iron and zinc are the most abundant metals in cells, followed by copper and manganese, and, to a lesser extent, cobalt, nickel, and molybdenum. Thus, it is not surprising to discover the existence of intracellular systems for the distribution of metals and metal cofactors. Why do cells need these metal delivery systems? It is because the cell faces several obstacles in achieving the two major goals of metal cofactor delivery, which are incorporation of the native cofactor and exclusion of non-native cofactors.

A major obstacle to acquisition of metal cofactors by apoenzymes is that the binding sites for these cofactors frequently lack the capacity to discriminate between different divalent metal cations and cofactors (Kasampalidis et al., 2007; Waldron et al., 2009). The sulfur, oxygen, and nitrogen ligands that coordinate metals in enzymes will frequently bind non-native cofactors with affinities equal to or greater than those of the native cofactor. A second obstacle is that redox-active metals, such as iron, copper, and manganese can engage in Fenton-type chemistry in the presence of oxygen and produce potentially damaging reactive oxygen species. Thus, cells must tightly regulate the uptake, storage, and distribution of metal ion species. A third obstacle is that zinc and copper ions exhibit the highest affinity for transition metal-binding sites and would occupy iron and manganese sites if the metals were present in freely exchangeable pools of similar concentrations (Irving and Williams, 1953). Consequently, cells maintain pools of zinc and copper at exceedingly low levels (Rae et al., 1999; Outten and O’Halloran, 2001). This is accomplished through tightly regulated uptake and efflux and through the expression of metal-binding proteins, such as metallothioneins, that effectively sequester pools of zinc and copper. Iron, however, appears to be managed differently.

## THE FATE OF INTRACELLULAR IRON

**FIGURE 1:**
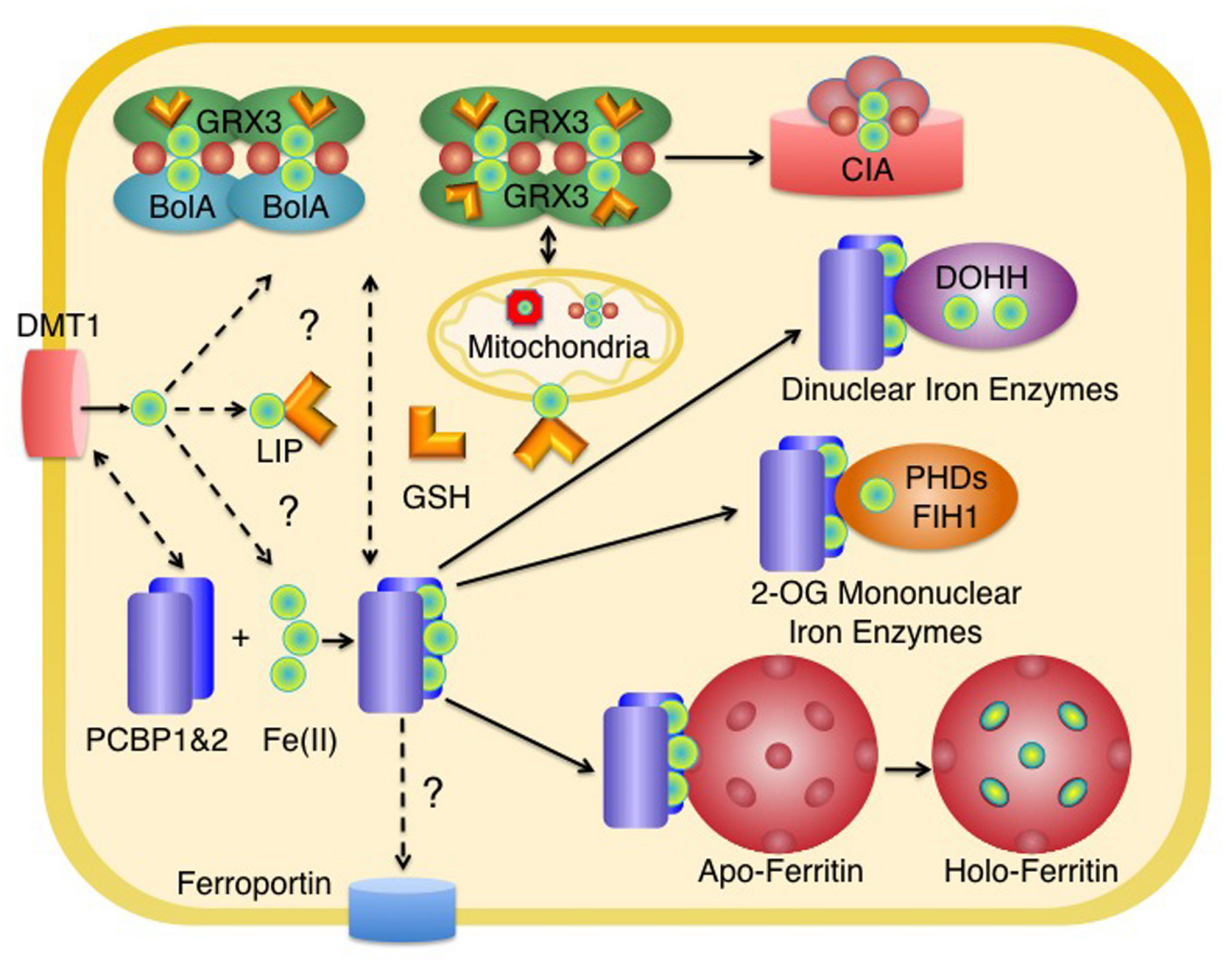
**Distribution of iron in the cytosol of mammalian cells.** Iron enters the cytosolic labile iron pool (LIP) as Fe(II) and may be coordinated by reduced glutathione (GSH). Most of the iron entering the LIP is delivered to mitochondria for the synthesis of heme and Fe–S clusters. In the cytosol, homodimers of Grx3 or heterooligomers of Grx3 and BolA2 coordinate [2Fe-2S] clusters with the help of GSH. These clusters may be used for the metallation of cytosolic Fe-S enzymes (via the cytosolic iron–sulfur cluster assembly machinery, CIA) and may have a role in mitochondrial iron delivery in erythroid tissues. PCBP1 and PCBP2 bind iron in the cytosol and deliver it to target non-heme iron enzymes, such as ferritin, 2-oxoglutarate-dependent dioxygenases (mononuclear iron enzymes such as PHD2 or FiH1), and oxo- or peroxo-bridged diiron oxygenases (dinuclear iron enzymes such as DOHH). The source of iron for PCBPs is not known, but could include components of the LIP coordinated by GSH or iron coordinated by Grx3. PCBP2 may directly acquire iron from transporters such as DMT1 and may also deliver iron to ferroportin for efflux. Some intracellular organelles have been omitted for clarity. Red spheres indicate inorganic sulfide as part of an Fe–S cluster.

In mammals, iron enters the cell through a variety of transport systems, including the endosomal transporter DMT1 (**Figure 1**) (reviewed in Shawki et al., 2012), which receives transferrin-bound iron, and Zip14, which can take up both endosomal transferrin- and extracellular non-transferrin-bound iron (Liuzzi et al., 2006; Zhao et al., 2010). The majority of iron taken up through membrane transporters enters a metabolically active pool in the cytosol (Shvartsman and Ioav Cabantchik, 2012), although a small amount may be transferred directly from endosomes to mitochondria (Sheftel et al., 2007). The molecular nature of this cytosolic pool is largely unknown, but operationally it is defined as the freely exchangeable iron that is loosely coordinated by water, small molecules, and proteins, and has been termed the labile iron pool (LIP). Most of the iron entering the LIP is directed to mitochondria (Shvartsman and Ioav Cabantchik, 2012), where it can be incorporated into mononuclear or dinuclear iron centers, inserted into protoporphyrin IX to form heme, or used in the assembly of iron–sulfur clusters. Mitochondrial heme and iron–sulfur clusters are bound by enzymes within mitochondria, but heme and some product of the Fe–S cluster assembly machinery are also exported from mitochondria for utilization in the cytosol, nucleus, and other membrane-bound organelles. Cytosolic iron that is not directed to mitochondria may be used to metallate non-heme iron enzymes of the cytosol and nucleus, to assemble cytosolic Fe–S clusters, or be stored and sequestered in ferritin. The various fates of cytosolic iron are likely determined by the activity of iron transporters, such as the mitochondrial iron importers (Mitoferrin 1 and Mitoferrin 2, Shaw et al., 2006) and iron efflux pumps on the lysosomal or plasma membrane (e.g., ferroportin, Abboud and Haile, 2000; Donovan et al., 2000; McKie et al., 2000). Iron storage in ferritin is largely determined by the level of ferritin protein, which is tightly regulated according to cellular iron levels (reviewed in Anderson et al., 2012). The partitioning of iron to iron-binding small molecules and proteins of the LIP and proteins specialized for the delivery of iron cofactors to recipient apoenzymes play important roles in determining the fate of cytosolic iron.

## COMPOSITION OF THE CYTOSOLIC LABILE IRON POOL

From a conceptual standpoint, the cytosol must contain a kinetically labile, metabolically accessible pool of iron so that iron can be distributed to its various sites of utilization, transport, or storage (reviewed in Hider and Kong, 2013). Technical hurdles have hampered the study of the LIP largely because of the need to study the pool in intact, living cells. Breaking open cells, diluting their contents, and exposing the contents to oxygen will greatly alter the character of the LIP. From a practical standpoint, the measurable LIP is defined as the pool of iron that is accessible to weak iron chelators introduced to the cytosol (Petrat et al., 2002). Fluorescent iron chelators are used to measure the LIP (Esposito et al., 2002). Chemically, these compounds are composed of a fluorescein moiety conjugated to an Fe(II)- or Fe(III)-binding moiety. These chelators are introduced to cells as a lipophilic, esterified precursor, which is then hydrolyzed and trapped in the cytosol. Iron binding quenches fluorescence; when a cell-permeable, strong iron chelator is then added, iron shifts from the fluorescent chelator to the strong chelator and fluorescence is regained. The ratio of fluorescence after and before addition of the strong iron chelator provides an estimate of the chelatable iron pool. Using this approach, the cytosolic LIP has been measured with calcein and found to be 0.3–1.6 μM, with different cell types exhibiting different baseline levels of the LIP and different responses to iron-supplemented or iron-depleted medium. Measurements using Phen Green-SK suggest higher levels for the cytosolic LIP, which is consistent with the much higher affinity of Phen Green-SK for Fe (II). Measurements with more iron-specific fluorescent chelators also indicate the LIP to be in the low micromolar range. Intracellular iron species in whole cells have also been measured using spectroscopic methods. Human Jurkat cells grown in iron-supplemented medium were found to contain ∼30 μM high-spin Fe(II) in the cytosol, a portion of which likely represents the LIP, with the remainder coordinated by non-heme iron enzymes (Jhurry et al., 2013).

Attempts have been made to characterize the molecular composition of the cytosolic LIP and some progress has been made. Several approaches based upon the relative binding of iron species to Fe(II)- and Fe(III)-specific chelators before and after treatment with reductants or oxidants indicate that >80–90% of the cytosolic LIP is in the reduced state (Egyed and Saltman, 1984; Breuer et al., 1995). Using Mossbauer and electron paramagnetic resonance spectroscopy, the cytosolic iron pool of mammalian cells was found to contain high-spin Fe(III) signals typical of ferritin and Fe(II) signals in the form of heme and non-heme iron (Jhurry et al., 2013). This predominance of Fe(II) is consistent with the presence of excess reduced glutathione and NADPH in the cytosol, both of which can act as physiologic iron reductants at the pH and ionic strength of the cytosol. Furthermore, all known metazoan iron uptake systems are specific for Fe(II). Thus, the iron initially entering the cytosol is also in the reduced state.

## GLUTATHIONE AS A MAJOR CYTOSOLIC LIGAND FOR Fe(II)

Because the cytosolic LIP is essentially present as Fe(II), the physiologic ligands for the cytosolic LIP must have affinity for Fe(II). Many small molecules proposed as physiologic ligands are unlikely to form complexes with cytosolic iron because they form stable complexes with Fe(III) and not Fe(II). These would include ATP/AMP, free amino acids, inositol triphosphates (Veiga et al., 2009), and 2,5 dihydroxybenzoic acid (Devireddy et al., 2010; Shvartsman and Ioav Cabantchik, 2012). Candidates for physiologic Fe(II) ligands include citrate, cysteine, and reduced glutathione (GSH). Analysis of these potential ligands *in vitro* suggests that Fe–GSH complexes are likely the only species that forms at significant levels in cells, largely because GSH is present at relatively high (2–10 mM) levels in the cytosol and exhibits moderate affinity (*K*_d_∼8 μM) for Fe(II) (Hider and Kong, 2011). Other ligands that demonstrate strong affinities for Fe(II) may not form complexes because they are present at low concentrations in the cytosol. Genetic studies in yeast support an important role of Fe-GSH complexes in the LIP. In yeast cells engineered to overexpress a GSH importer, GSH overload was associated with activation of the iron-sensing transcription factor Aft1 and impaired activity of a cytosolic iron–sulfur cluster enzyme (Kumar et al., 2011). Depletion of cellular GSH by deletion of the essential γ-glutamylcysteine synthetase also led to Aft1 activation and impaired cytosolic Fe–S cluster enzyme activity, which could be partially restored with exogenous iron (Sipos et al., 2002; Kumar et al., 2011); neither of these phenotypes was due to oxidative stress or damage. These studies point to Fe–GSH complexes as having a critical role in maintaining iron distribution and homeostasis in eukaryotic cells. Additional studies indicate that Fe–GSH, in conjunction with monothiol glutaredoxins, has a direct role in the formation and transfer of cytosolic Fe–S clusters.

## PROTEINS INVOLVED IN CYTOSOLIC IRON DISTRIBUTION: MONOTHIOL GLUTAREDOXINS

In addition to its role as a ligand in the cytosolic LIP, GSH coordinates iron as part of the Fe–S carrier complex formed by cytosolic monothiol glutaredoxins (reviewed in Rouhier et al., 2010). The glutaredoxins are a ubiquitous class of enzyme with a thioredoxin fold and are generally thought to function as thiol-disulfide oxidoreductases with roles in glutathione conjugation. These enzymes contain one or two cysteine residues in their active site that are critical for enzymatic activity. A subset of the glutaredoxin family does not exhibit oxidoreductase activity and contains a monothiol active site motif that consists of the tetrapeptide CGFS. These monothiol glutaredoxins instead function as Fe–S cluster carriers. Eukaryotic cells contain separate monothiol glutaredoxins that localize to either the mitochondria (Grx5) or the cytosol (Grx3/4). Grx5 plays an important role in the assembly and transfer of Fe–S clusters in mitochondria (Rodriguez-Manzaneque et al., 2002; Wingert et al., 2005; Ye et al., 2010). Yeast, fish, and human cells lacking Grx5 exhibit profound defects in the assembly of both mitochondrial and cytosolic Fe–S clusters. Recent evidence suggests that cytosolic Grx3-type glutaredoxins may have a broader role in cytosolic iron distribution.

Structurally, Grx3-type glutaredoxins have a single N-terminal thioredoxin domain and 1–3 C-terminal glutaredoxin domains (Rouhier et al., 2010). The functionally interchangeable Grx3 and Grx4 of yeast contain a single CGFS glutaredoxin domain while the sole human ortholog, Grx3 (also called Glrx3 and PICOT), contains two. *In vitro*, Grx3 forms homodimers that can accommodate a bridging [2Fe-2S] cluster, which is coordinated by the single active site cysteine present in each monomer and by the free sulfhydryl on each of two molecules of GSH. The cluster is labile, sensitive to both reductants and oxidants, and can be transferred to a recipient Fe–S scaffold protein (Li et al., 2009; Haunhorst et al., 2010).

In many species, including prokaryotes, plants, yeast, and metazoans, monothiol glutaredoxins may be found in complex with BolA-like proteins, with distinct complexes formed in the cytosol/nucleus, mitochondria, and plastids. *In vitro*, Grx3 domains can form heterooligomers with the yeast and human cytosolic BolA2-type proteins in a 1:1 stoichiometric ratio. These complexes also coordinate bridging [2Fe-2S] clusters, with BolA2 contributing a histidine ligand to coordinate the cluster(s) (Li et al., 2011). Clusters formed on Grx3–BolA2 complexes exhibit more stability than those formed on Grx3 homodimers, and Grx3 homodimers will spontaneously transfer a [2Fe-2S] cluster to Grx3–BolA2 *in vitro* (Li et al., 2009).

Genetic studies in yeast, fish, and human cells suggest that Grx3 orthologs have similar but distinct roles in cytosolic iron delivery. In yeast, assembly of Fe–S clusters on Grx4 requires the cytosolic Fe–S assembly machinery, but it is not dependent on the remaining cytosolic Fe–S cluster machinery. Although Grx3/4 is expressed in the cytosol and nucleus, their activities affect iron handling in both the cytosol and mitochondria. Strains lacking Grx3/4 exhibit defects in the incorporation of iron–sulfur clusters into mitochondrial Fe–S enzymes (fivefold decrease in aconitase), defects in the incorporation of iron into heme (fivefold decrease), and reduced accumulation of iron into mitochondria (2.3-fold decrease). These observations are all consistent with a defect in mitochondrial iron delivery. In the cytosol, Grx3/4-deficient strains also exhibit reduced metallation of Fe–S enzymes (four to tenfold decrease). Furthermore, a cytosolic dinuclear iron enzyme, ribonucleotide reductase, also appears to require Grx3/4 for acquisition of iron (Muhlenhoff et al., 2010). This study suggests that Grx3/4 has roles in the delivery of iron to mitochondria and to cytosolic non-heme enzymes as well as in the delivery or assembly of Fe–S clusters.

Grx3-type glutaredoxins from several species have been found to interact with BolA-like proteins *in vivo*. In yeast, Grx3/4 and Fra2, the cytosolic BolA ortholog, have been found together in a complex with Aft1, the major iron-dependent transcription factor (Kumanovics et al., 2008). Grx3/4-Fra2 complexes appear to have a specialized regulatory function in yeast, as both are required for the sensing of iron (Ojeda et al., 2006; Pujol-Carrion et al., 2006) and can mediate the transfer of a [2Fe-2S] cluster to the Aft1 paralog, Aft2 (Poor et al., 2014). Zebrafish contain a single Grx3 ortholog, which could be depleted in embryos through morpholino injection. Zebrafish embryos lacking Grx3 exhibited severe defects in heme synthesis in tissues devoted to embryonic erythropoiesis (38% of embryos exhibited hemoglobin staining), but mild defects in the activities of Fe–S cluster and heme enzymes (20% decrease). In human cells, knockdown of Grx3 was associated with loss of Fe–S cluster-dependent, cytosolic aconitase activity (60% decrease) and concomitant disruption of cellular iron homeostasis. In contrast, activities of mitochondrial heme enzymes and heme synthesis were only mildly diminished (20% decrease, Haunhorst et al., 2013). Thus, in vertebrates, Grx3 may be important for iron delivery to mitochondria primarily in erythropoietic tissues, which have very high requirements for iron. Grx3 may also be involved in cytosolic Fe–S cluster delivery or assembly. Although *in vitro* studies have demonstrated that Grx3–Fra2 complexes can donate a [2Fe-2S] cluster to Aft2, and genetic studies in yeast support this evidence of a direct Fe–S transfer to Aft1, studies have not yet made clear whether Grx3-type homodimers directly deliver Fe–S clusters or iron ions to targets in yeast or human cells. Similarly, the role of Fra2/BolA2 in human cells is largely unknown, although the mitochondrially targeted BolA3 is involved in Fe–S cluster acquisition and/or maintenance for some mitochondrial Fe–S enzymes, especially lipoate synthase (Cameron et al., 2011; Haack et al., 2013; Baker et al., 2014). The mechanism by which Grx3 acquires iron is also not yet known, but it appears not to require the activity of the cytosolic Fe–S cluster assembly system.

## PROTEINS INVOLVED IN CYTOSOLIC IRON DISTRIBUTION: PCBPs

The term metallochaperone has been used to describe proteins that directly deliver metals to target enzymes or transporters through metal-mediated protein–protein interactions (Lee et al., 1993; Culotta et al., 1997; Pufahl et al., 1997). Iron chaperone activity has been demonstrated for the poly C binding protein (PCBP) family of proteins (Shi et al., 2008). PCBP1 was initially identified as an iron chaperone for ferritin, the ubiquitous iron storage protein. Ferritin, composed of 24 subunits of H- and L-isoforms, functions as a cellular repository of surplus iron by accommodating up to 4500 iron atoms within its spherical core. Delivery of iron atoms into the hollow sphere of ferritin occurs via the hydrophilic channels formed by the carboxylate side chains along the threefold symmetry axes in the heteropolymer. Initially, ferritin accrues iron atoms in the ferrous form, which are then oxidized to ferric iron by the ferroxidase center of H-ferritin, located in the interior of the ferritin cavity (reviewed in Arosio and Levi, 2010). Even though the regulatory mechanisms of this gene product have been extensively studied under various physiological conditions, the cytosolic trafficking system directing iron to the metalloprotein had been elusive until the recent discovery of the iron chaperone activity of PCBP1.

The role of PCBP1 in the mineralization of ferritin was initially identified through functional screening of a human liver cDNA library in a eukaryotic model lacking endogenous ferritin expression, i.e., *Saccharomyces cerevisiae* (Shi et al., 2008). Co-expression of human ferritins and PCBP1 in yeast cells produced an iron deficiency response, implying sequestration of cellular iron into the exogenous iron storage protein. This was confirmed by measuring PCBP1-dependent increases in ferritin iron content. The requirement of endogenous PCBP1 in mammalian cells was shown by loss-of-function experiments using cultured human Huh7 cells. RNA interference of PCBP1 led to inefficient mineralization of ferritin (63% reduction) along with increases in iron-mediated degradation of iron-regulatory protein 2 (IRP2) and in the cellular LIP (67% increase). *In vitro* experiments with purified PCBP1 and ferritin supported the direct involvement of PCBP1 in the mineralization of ferritin by a dose-dependent enhancement in the incorporation of iron into apoferritin by the chaperone protein.

PCBP1 [also known as heterogeneous nuclear ribonucleoprotein (hnRNP) E1 or α-CP1] is a member of the poly(rC)-binding protein family, composed of four homologous proteins of which others are PCBP2, PCBP3, and PCBP4. PCBPs were initially characterized as RNA-binding proteins, modulating the stability or translation of cellular and viral RNA species containing C-rich motifs (Makeyev and Liebhaber, 2002; Ostareck-Lederer and Ostareck, 2004; Chaudhury et al., 2010). Other regulatory functions include their involvement in transcriptional regulation of gene expression and protein–protein interactions. PCBPs contain three highly conserved hnRNP K homology (KH) domains, which are ancient, RNA-binding motifs broadly distributed in prokaryotes and eukaryotes. Sequences outside of the KH domains exhibit much less conservation. PCBP1 and PCBP2 are expressed at high levels in essentially all mammalian cell types, while PCBP3 and PCBP4 exhibit much lower levels of expression in a limited range of tissues. PCBP1 is specific to mammals, while genes orthologous to those of PCBP2, 3, and 4 are present in vertebrates, flies, and worms, and distantly related orthologs are present in yeast.

Recently, PCBP2 was also identified as an iron chaperone for ferritin, conferring increased iron storage when co-expressed in yeast and manifesting decreased iron storage when depleted from human Huh7 cells (Leidgens et al., 2013). PCBP3 could also induce iron deficiency responses in yeast by enhancing the sequestration of iron into exogenously expressed ferritin. Notably, the presence of KH domains alone was not sufficient for conferring iron delivery activity to PCBPs, as a splice variant of PCBP3 lacking 26 amino acids between KH domains 2 and 3 and an unrelated RNA-binding protein containing two KH domains (fragile X mental retardation 1) had essentially no iron activity in yeast cells. PCBP4 also activates the iron deficiency response in yeast, but it shows weak genetic and physical interactions with ferritin. PCBP4 could function as a buffer for labile iron or could be involved in organellar iron delivery in specialized cell types.

Purified recombinant PCBP1 and PCBP2 were found to bind ferrous iron *in vitro* using isothermal titration calorimetry (ITC). ITC characterizes the thermodynamic properties of a ligand binding reaction, and thus allows a quantitative measurement of both binding affinity and stoichiometry. Anaerobic titration of ferrous iron into purified PCBP1 revealed an iron-binding capacity of three ferrous iron atoms per molecule of PCBP1, with a dissociation constant of 0.9 ± 0.1 μM for the first and an average of 5.8 ± 0.3 μM for the remaining two (Shi et al., 2008). PCBP2 exhibits similar iron-binding characteristics (T. Stemmler, personal communication). Physical interactions between PCBP1, PCBP2, and ferritin were also measured using ITC (Leidgens et al., 2013). Purified PCBPs exhibited no significant interaction with ferritin in the absence of iron. However, PCBP1 and PCBP2, anaerobically loaded with ferrous iron, exhibited affinities for apoferritin that were 30- and 20-fold higher, respectively, than the affinity of free ferrous iron for ferritin. Approximately, nine Fe-PCBP1 molecules bind to a ferritin oligomer. As the number of putative iron delivery channels is eight per ferritin polymer, this stoichiometry supports a model of PCBP1 facilitating iron incorporation into ferritin via direct binding at pores formed by the threefold axes of symmetry. Fe-PCBP2 binds to ferritin in a stoichiometric ratio of 4:1 Fe-PCBP2:ferritin.

In human cells, PCBP1, PCBP2, and PCBP3 can be isolated in complexes with ferritin (Leidgens et al., 2013). PCBP1 and PCBP2 are also found to interact with each other, both in yeast and human cells. Two lines of evidence suggest that PCBP1 and PCBP2 may act together in iron delivery. First, Huh7 cells depleted of PCBP1 or PCBP2 contain wild-type levels of the other paralog, yet exhibit marked defects in ferritin iron loading. The simultaneous depletion of both PCBP1 and PCBP2 produces an iron storage defect similar in magnitude to the single deletion of either. These observations indicate that PCBP1 cannot substitute for PCBP2 in human cells and vice versa. Second, quantitative immunoprecipitation studies indicate that the interaction between ferritin and PCBP1 is diminished when PCBP2 is depleted; similarly, the co-precipitation of PCBP2 with ferritin is diminished in cells depleted of PCBP1. These observations suggest that PCBP1 and PCBP2 function cooperatively, perhaps as a complex, in the distribution of iron.

## METALLATION OF NON-HEME IRON ENZYMES

In addition to ferritin, PCBPs exhibit iron chaperone activity toward other cytosolic iron enzymes. The class of iron- and 2-oxoglutarate (2-OG)-dependent dioxygenases comprises a large family of non-heme iron enzymes that require the incorporation of a single iron atom into its active site for activation (Ozer and Bruick, 2007; Loenarz and Schofield, 2008). Recently, two of these mononuclear iron enzymes, prolyl hydroxylase 2 (PHD2) and the asparaginyl hydroxylase, factor inhibiting HIF1 (FIH1), have been identified as clients for iron delivery by PCBPs (Nandal et al., 2011).

Hypoxia-inducible factors (HIF) are heterodimeric transcription factors that mediate the cellular adaptation to hypoxia (Semenza, 2007; Kaelin and Ratcliffe, 2008). Conditions of reduced oxygen lead to the accumulation of the alpha subunits (HIFα), HIF1α and HIF2α, which assemble into the active form with its constitutively expressed counterpart, HIF1β. In normoxia or hyperoxia, PHD hydroxylates HIFα on proline residues within the highly conserved oxygen-dependent degradation domains. Upon recognition by the von Hippel-Lindau (VHL) tumor suppressor protein, hydroxylated HIFα undergo proteolysis via the ubiquitin-proteasome pathway. FIH1 also regulates HIFα via hydroxylation of a conserved amino acid, in this case, however, at asparagine. The hydroxylation at the asparaginyl residue blocks the interaction of the alpha subunits with coactivators and thus results in the inactivation of HIF. In the absence of oxygen, a co-substrate for both enzymes, HIFα subunits remain unmodified and can accumulate and assemble into active heterodimers.

The activities of the oxygen-sensing HIF hydroxylases are also responsive to the availability of iron. Treatment of cells with supplemental iron results in an increase in the HIF hydroxylase activity, while iron chelation produces the opposite effect (Kaelin and Ratcliffe, 2008). Moreover, induction of HIF2 signaling has been reported in the small intestine of mice subjected to dietary iron deficiency, which may be attributable to compromised HIF hydroxylase activity (Shah et al., 2009). While the requirement of iron for HIF hydroxylase activation has been known for several decades, the mechanism which mediates the incorporation of a mononuclear iron center into the apoenzyme remained unknown until recent studies implicated the involvement of PCBPs.

Cultured human cells lacking PCBP1 or PCBP2 exhibit nuclear accumulation of active HIF1α, which increases in cells made mildly or transiently iron deficient (Nandal et al., 2011). Loss of PCBP expression leads to prolonged HIF1α half-life without altering steady-state levels of HIF1α transcripts. The responses of HIF1α to PCBP1 or PCBP2 depletion are due to the inactivation of PHD2, which accounts for nearly all of the prolyl hydroxylation of HIF1α in cells (Berra et al., 2003). Cells depleted of PCBP1, PCBP2, or both exhibit reduced incorporation of the iron cofactor into PHD2 (20–40% of control) and complete loss of enzyme activity in cell lysates. In lysates, the effects of PCBP depletion on PHD activity can be reversed by exogenous addition of excess iron. As for the case of ferritin, PCBP1 can form complexes with PHD2 in human cells. Thus, the PCBP-mediated delivery of iron into the active site of the mononuclear iron enzyme is likely accomplished via direct protein–protein interaction.

The activity of FIH1, also an iron- and oxygen-dependent HIF1α regulator, is dependent on PCBP1 as well (Nandal et al., 2011). Cells depleted of PCBP1 exhibit increased expression of a reporter construct repressed by FIH1, and FIH1 was detected in a complex with PCBP1 in iron-treated cells. The loss of FIH1 activity and the physical interaction between cellular PCBP1 and FIH1 suggest that FIH1 is also a target for iron delivery by PCBP1. Whether all iron- and 2-OG-dependent enzymes require PCBPs for their metallation and activation remains undetermined.

Other classes of iron metalloenzymes may also depend on iron delivery by PCBPs. Of particular interest are the dinuclear, non-heme iron enymes, some of which contain iron-binding active sites structurally related the ferroxidase site of ferritin, a known target of PCBPs. Examples of this class of enzymes are the small subunit of ribonucleotide reductase (Stubbe and Cotruvo, 2011) and deoxyhypusine hydroxylase (DOHH, Vu et al., 2009). DOHH is a dinuclear iron enzyme that is required for the posttranslational modification of a single lysine residue on eukaryotic initiation factor 5A (eIF5A, Park et al., 2010). EIF5A and the conversion of this conserved lysine to hypusine are essential in all eukaryotes, as it enables the translation of peptides containing polyproline sequences (Gutierrez et al., 2013). Cells lacking PCBP1 or PCBP2 exhibit loss of DOHH activity, as measured by the accumulation of partially modified deoxyhypusine and reduced levels of fully modified hypusine (20% of control) in living cells. DOHH from PCBP-depleted cells also exhibits loss of the iron cofactor when the cells are exposed to mild iron deficiency. As for other PCBP targets, DOHH is detected in a complex with PCBP1, indicating their direct association during the assembly of the diiron active site. PCBPs do not appear to be required for iron delivery to mitochondria or for the *de novo* assembly of iron–sulfur clusters in the cytosol (Frey et al., 2014). Collectively, PCBPs can regulate the cellular distribution of labile iron by depositing it into the cellular iron reservoir ferritin and/or by directing it to the site of utilization as an inorganic cofactor for cytosolic non-heme iron enzymes.

## ROLE OF PCBPs IN REGULATION OF IRON HOMEOSTASIS

The iron chaperone activities of PCBPs do not appear to be regulated in response to changing cellular iron availability. That is, the expression level and iron-binding activity do not appear to change under different iron conditions (Frey et al., 2014). Although interactions with client enzymes do appear, in some cases, to be lessened when iron is scarce, this effect could be mediated solely by the lessened stability of the chaperone–client interaction in the absence of bound iron. PCBPs may, however, influence cellular iron homeostasis through their iron chaperone activities. Huh7 cells depleted of PCBPs store less cytosolic iron in ferritin, thereby expanding the LIP (Shi et al., 2008). This expansion of the LIP is reflected in lowered levels of iron regulatory protein 2 (IRP2), which undergoes ubiquitin-mediated degradation in the presence of increased labile iron (Guo et al., 1995). Whether the degradation associated with PCBP depletion is dependent on the recently described ubiquitin ligase FBXL5 has not been determined (Salahudeen et al., 2009; Vashisht et al., 2009). HEK 293T cells depleted of PCBPs exhibit reduced activity of cytosolic (c-) aconitase (Frey et al., 2014), an enzyme that carries a labile [4Fe-4S] cluster in its active site. C-aconitase, also called iron regulatory protein 1 (IRP1), is a bifunctional enzyme because it acquires RNA-binding activity when the [4Fe-4S] cluster is absent (reviewed in Anderson et al., 2012). Together, IRP1 and IRP2 regulate cellular iron uptake and storage by altering the translation and stability of mRNA transcripts that contain iron-responsive elements (IREs) in the 5′ or 3′ UTR. These transcripts encode proteins of iron uptake, e.g., transferrin receptor and divalent metal transporter 1 (DMT1), and iron storage, e.g., ferritin. Although PCBP depletion leads to loss of c-aconitase activity, it does not appear to lead to a reciprocal increase in IRE-binding activity, suggesting that PCBP depletion does not result in the complete disassembly of the Fe–S cluster in IRP1. In fact, PCBP depletion was associated with loss of IRP1-mediated IRE binding activity. Thus, loss of cellular PCBPs results in loss of IRP1- and IRP2-mediated IRE-binding activity, which likely produce misregulation of proteins that maintain the iron balance of the cytosol.

Tissues may also exhibit specific requirements for PCBPs in the delivery of iron. In mice, dietary iron deficiency leads to increased HIF2α in intestinal epithelial cells, where it promotes the transcription of DMT1 and other genes involved in the absorption of dietary iron (Mastrogiannaki et al., 2009; Shah et al., 2009). Intestinal HIF2α is likely regulated by prolyl and asparagyl hydroxylases, which receive iron cofactors from PCBP1 and PCBP2. Thus, PCBPs may be important for the response to iron deficiency in gut cells. Recently, H-ferritin was determined to be an indispensible factor for accurately controlling intestinal iron absorption, primarily by retaining iron in absorptive enterocytes and preventing excessive iron transfer from enterocytes to the systemic circulation (Vanoaica et al., 2010). Loss of H-ferritin expression in the small intestine led to characteristic manifestations of hemochromatosis. The perturbation in systemic iron homeostasis was attributed to higher systemic absorption of iron due to enhanced efflux from enterocytes through the basolateral transporter, ferroportin 1. Dysfunction of the iron storage protein also affected cellular iron homeostasis in the enterocytes. It is not yet known whether PCBPs mediate ferritin iron loading in the intestinal enterocyte, but if enterocytes do rely on PCBPs, they may also serve as a limiting component for body iron accrual by directing and loading the metal to ferritin. In addition to these indirect effects on iron uptake, storage and efflux, PCBPs may exert direct effects on transport. A recent report indicates that PCBP2 directly interacts with both DMT1 and ferroportin in cells, enhancing the transport of iron via DMT1 (Yanatori et al., 2014). Other cell types, such as macrophages and erythropoietic cells have special requirements for iron handling, as they accommodate the large iron fluxes associated with the recycling of iron from senescent red blood cells and hemoglobin synthesis, respectively. Whether PCBPs play a role in these processes remains to be determined. An important consideration in these studies is the kinetics involved in the intracellular iron delivery systems. What is the time frame for delivery of iron and metallation of enzymes in the cell? New approaches will be required to address these types of questions. In summary, PCBPs may provide strategies for the efficient management of the newly acquired or mobilized iron atoms by efficiently delivering them to their appropriate destination, particularly in cell types that routinely encounter dynamic changes in the cytosolic LIP.

## Conflict of Interest Statement

The authors declare that the research was conducted in the absence of any commercial or financial relationships that could be construed as a potential conflict of interest.
